# Reimagining the Community Nursing Bag: Eco-Smart and Technology-Integrated Solutions

**DOI:** 10.7759/cureus.112333

**Published:** 2026-07-09

**Authors:** S Niranjani, Prema Krishnan, T Umadevi, Jayamalli N

**Affiliations:** 1 Community Health Nursing, Shri Sathya Sai College of Nursing, Sri Balaji Vidyapeeth, Chennai, IND; 2 Child Health Nursing, Shri Sathya Sai College of Nursing, Sri Balaji Vidyapeeth, Chennai, IND; 3 Nursing, Shri Sathya Sai College of Nursing, Sri Balaji Vidyapeeth, Chennai, IND; 4 Nursing, Sri Ramaswamy Memorial (SRM) Institute of Science and Technology, Chennai, IND

**Keywords:** community health nursing, community nursing bag, digital health, eco-smart healthcare, infection prevention, sustainability, technology integration, telehealth

## Abstract

Community health nurses play a vital role in delivering preventive, promotive, curative, and rehabilitative healthcare services through home visits and outreach programs. The community nursing bag has long served as an essential tool for carrying supplies, equipment, medications, and records required for field-based nursing care. However, conventional community bags face several limitations, including infection-control concerns, excessive weight, poor ergonomic design, environmental impact, and limited integration with digital technologies. These challenges necessitate the modernization of traditional community nursing bags to meet the evolving demands of contemporary healthcare practice.

This review explores the concept of eco-smart and technology-integrated community nursing bags as innovative solutions for enhancing field-based nursing care. This review discusses the traditional community bag technique, existing practices, and major challenges associated with conventional bags. It further examines eco-smart innovations such as sustainable fabrics, reusable sterilizable kits, biodegradable packaging, solar-powered charging systems, and water-resistant eco-friendly materials that promote environmental sustainability and improve usability. Technological advancements, including portable diagnostic devices, digital documentation systems, telehealth connectivity, Global Positioning System (GPS) technology, artificial intelligence (AI), and Internet of Things (IoT)-enabled applications, are highlighted for their potential to improve assessment, communication, continuity of care, and patient monitoring. This review also addresses infection-prevention enhancements such as antimicrobial surfaces, UV sterilization units, contamination-control compartments, and integrated hand hygiene systems. The relevance of these innovations to rural healthcare, disaster response, maternal and child health services, and geriatric home care is discussed. Additionally, challenges related to cost, training, digital literacy, and infrastructure limitations are examined. Future directions emphasize policy support, curriculum reform, research, and prototype development. Reimagining the community nursing bag as an eco-smart and technology-enabled system has the potential to improve healthcare accessibility, patient safety, sustainability, and quality of care, thereby strengthening community and public health nursing practice in the digital era.

## Introduction and background

Community health nursing plays a crucial role in delivering preventive, promotive, curative, and rehabilitative healthcare services directly to individuals, families, and communities. The community bag is a fundamental tool in field-based nursing practice, serving as a portable unit that contains essential supplies, equipment, records, and emergency materials required for home visits and outreach services [1]. Traditionally, the community bag has enabled nurses to provide organized, safe, and effective care while maintaining infection-prevention practices and continuity of healthcare services. Over the years, the community bag has supported a wide range of community health activities, including maternal and child healthcare, immunization, school health services, chronic disease management, geriatric care, and health education. However, evolving healthcare demands have highlighted several limitations of conventional community bags, including inadequate organization, infection-control challenges, excessive weight, environmental concerns, and limited integration with digital technologies [2]. Recent advancements in telehealth, portable diagnostics, electronic health records, wearable devices, and artificial intelligence have transformed community healthcare delivery, while the increasing emphasis on sustainability has encouraged the adoption of eco-friendly and reusable healthcare solutions [3,4]. Consequently, there is a growing need to modernize traditional community bags by incorporating sustainable materials, smart technologies, improved infection-control features, and digital connectivity. This narrative review explores the significance, challenges, innovations, and future prospects of eco-smart and technology-integrated community bags for enhancing field-based nursing practice.

## Review

Traditional community bag technique and existing practices

The traditional community bag technique is a systematic method used by community health nurses during home visits to provide safe, organized, and effective nursing care while preventing the transmission of infection [5]. The community bag serves as a portable nursing unit containing essential equipment, medicines, sterile supplies, infection-control materials, and records required for field-based healthcare delivery [1]. Its primary purpose is to facilitate health assessment, treatment, health education, emergency care, and continuity of care in homes, schools, industries, and community settings [6].

A typical community bag contains assessment tools such as a stethoscope, blood pressure apparatus, and thermometer; sterile supplies including gloves, dressings, and syringes; medicines and antiseptics; laboratory materials; hand hygiene items; and documentation records [7]. Traditionally made of durable canvas, leather, or plastic, the bag is designed with multiple compartments for systematic organization and easy accessibility of supplies. The technique promotes aseptic practices, efficient service delivery, preparedness for emergencies, family-centered care, and prevention of cross-infection, while also symbolizing professionalism and accountability in community health nursing [1].

Current practices emphasize maintaining cleanliness, proper hand hygiene, segregation of clean and contaminated items, use of protective barriers during procedures, routine disinfection of reusable items, and regular restocking of supplies [5]. Despite its continued importance, conventional community bags rely heavily on manual documentation, bulky equipment, and traditional materials. Concerns regarding excessive weight, limited ergonomic design, and lack of digital integration have highlighted the need for modernization. With the increasing use of portable diagnostics, telehealth, electronic health records, and sustainable healthcare approaches, there is a growing demand for eco-smart and technology-integrated community bags that can support efficient, safe, and contemporary field-based nursing practice [2].

Challenges with conventional community bags

Conventional community nursing bags face several challenges that limit their effectiveness in modern healthcare practice. A major concern is the risk of infection transmission due to improper segregation of sterile and contaminated items, inadequate disinfection of reusable materials, poor hand hygiene, and placement of bags on contaminated surfaces during home visits [7,8]. Traditional bags also lack antimicrobial surfaces and dedicated compartments for biohazard disposal, increasing the risk of cross-contamination. Another significant challenge is weight and portability. Conventional bags are often bulky and heavy because they contain numerous supplies, records, and instruments, leading to fatigue, musculoskeletal discomfort, and reduced work efficiency, particularly among nurses working in rural and remote areas [2]. Limited ergonomic features further affect ease of transportation and organization. Lack of digital integration is another limitation, as traditional community bags rely largely on paper-based documentation and do not support electronic health records, telehealth technologies, mobile health applications, or portable digital devices [3]. This may reduce efficiency, delay decision-making, and hinder continuity of care. Environmental sustainability is also a concern because conventional bag practices involve extensive use of disposable plastics and non-biodegradable materials, contributing to biomedical waste and environmental pollution [4]. Additionally, inefficient compartmentalization and limited storage organization may delay procedures, compromise aseptic practices, and reduce operational efficiency during field visits [5]. These challenges highlight the need to redesign traditional community bags into eco-smart, lightweight, well-organized, and technology-integrated systems capable of meeting contemporary community healthcare demands.

Eco-smart innovations in community nursing bags

Eco-smart innovations aim to improve sustainability, durability, infection prevention, and environmental responsibility in community nursing practice. Modern community nursing bags are increasingly being designed using lightweight, durable, and eco-friendly materials such as recycled polyester, thermoplastic polyurethane (TPU)-coated fabrics, plant-based polymers, and polyvinyl chloride (PVC)-free alternatives. These materials provide water resistance, ease of disinfection, durability, and reduced environmental impact while enhancing portability and user comfort [9]. Reusable sterilizable kits are another important innovation that helps reduce biomedical waste generated by disposable medical supplies. Components such as reusable trays, sterilizable pouches, washable compartments, and autoclavable containers support sustainable and cost-effective healthcare delivery while maintaining infection-control standards. Similarly, biodegradable packaging made from plant-based materials, including polylactic acid (PLA), starch-based polymers, and biodegradable bioplastics, can significantly reduce plastic waste generated during home visits, immunization programs, and public health outreach activities [10]. Solar-powered charging systems represent a sustainable solution for community nurses working in rural, remote, and disaster-affected areas. Portable solar chargers can provide uninterrupted power for mobile phones, tablets, glucometers, pulse oximeters, and telehealth devices, thereby enhancing preparedness and healthcare accessibility in low-resource settings. In addition, water-resistant eco-materials such as TPU-coated fabrics, recyclable polyethylene, and Tyvek-based materials improve durability, contamination resistance, and longevity of nursing bags under challenging environmental conditions [11]. Collectively, these eco-smart innovations promote sustainable healthcare practices, reduce environmental burden, improve infection prevention, and enhance the efficiency and usability of community nursing bags in contemporary field-based nursing practice.

Technology integration in field nursing

Technological advancements have significantly transformed community health nursing by enhancing patient assessment, documentation, communication, and continuity of care. Modern community healthcare increasingly relies on portable digital devices that enable nurses to provide accurate, timely, and evidence-based care in homes and remote settings. Eco-smart community nursing bags are now being designed to accommodate digital blood pressure monitors, portable glucometers, smart thermometers, and pulse oximeters, which facilitate rapid point-of-care assessment and monitoring of chronic and acute health conditions. These devices support early detection and management of hypertension, diabetes mellitus, respiratory disorders, cardiovascular diseases, pregnancy-related complications, and other community health concerns [12]. Many modern diagnostic tools are lightweight, battery-operated, Bluetooth-enabled, and capable of storing patient data electronically, allowing healthcare providers to compare results over time and support teleconsultation services [3]. The integration of such technologies into community nursing bags improves clinical decision-making, reduces dependence on healthcare facilities for routine assessments, and enhances the efficiency and quality of field-based nursing care.

Digital documentation through tablets and mobile applications has transformed field nursing practice by enabling electronic health record management, real-time data entry, digital referrals, health education, and remote consultation support. QR code-based patient record systems further improve efficiency by allowing instant access to patient information, immunization records, and medical histories while reducing documentation errors and enhancing continuity of care. Global Positioning System (GPS)-enabled technologies assist nurses in household mapping, rural outreach planning, vaccination tracking, disaster response coordination, and epidemiological surveillance, thereby improving healthcare coverage and resource management [12].

Telehealth connectivity has emerged as a key component of modern community healthcare. Smartphones, tablets, portable internet systems, and cloud-based patient records enable community nurses to communicate with physicians and specialists in real time, improving healthcare accessibility and reducing unnecessary hospital visits, particularly in remote and underserved areas [3].

Furthermore, artificial intelligence (AI) and the Internet of Things (IoT) are increasingly being integrated into community healthcare systems. Smart reminder systems support medication adherence, follow-up visits, and immunization schedules, while automated inventory management systems help monitor stock availability and expiry dates of medical supplies. AI-supported triage systems assist nurses in identifying high-risk patients and prioritizing referrals based on real-time health data [13]. Remote patient monitoring devices, including wearable sensors, smartwatches, digital blood pressure (BP) monitors, continuous glucose monitors, and pulse oximeters, enable continuous monitoring of physiological parameters and facilitate early detection of clinical deterioration [14]. Overall, technology integration in community nursing bags enhances efficiency, accuracy, patient safety, healthcare accessibility, and continuity of care, making field-based nursing practice more responsive, connected, and patient-centered.

Infection prevention and safety enhancements

Infection prevention and patient safety are fundamental aspects of community health nursing. As community nurses frequently move between homes, clinics, schools, and outreach settings, the risk of cross-contamination through nursing equipment and supplies remains significant. Conventional nursing bags may serve as reservoirs for microorganisms if cleaning, disinfection, and proper bag management practices are not consistently followed [15]. To address these challenges, modern eco-smart nursing bags incorporate innovations such as antimicrobial surfaces, separate contaminated compartments, UV sterilization systems, and integrated hand hygiene facilities to reduce microbial transmission and improve patient safety.

Antimicrobial Surfaces

Modern nursing bags increasingly utilize antimicrobial-coated fabrics, silver-ion textiles, copper-based coatings, and antimicrobial polymers to reduce microbial contamination and healthcare-associated infections [16]. These materials help minimize bacterial growth, improve hygiene, and enhance the durability of reusable nursing bags. Studies have shown that nursing bags and frequently touched surfaces can harbor pathogenic organisms, including multidrug-resistant bacteria, increasing the risk of indirect transmission [15]. Although antimicrobial materials provide an additional layer of protection, regular cleaning and disinfection remain essential for effective infection prevention [17].

Separate Contaminated Compartments

Proper segregation of clean and contaminated materials is a key infection-control principle. Eco-smart nursing bags incorporate dedicated compartments for used instruments, biohazard waste, soiled dressings, sharps containers, and contaminated personal protective equipment (PPE). Color-coded, leak-proof, and sterilizable compartments help maintain aseptic practices and reduce cross-contamination during field procedures [18]. Such compartmentalized designs are particularly beneficial during wound care, maternal and child health visits, communicable disease management, and disaster-response situations.

UV Sterilization Units

Ultraviolet (UV-C) sterilization technology is emerging as an innovative infection-control solution for portable healthcare equipment. Compact UV sterilization units can be integrated into nursing bags to disinfect small instruments, thermometers, stethoscopes, pulse oximeters, mobile devices, and other frequently touched items. UV-based disinfection offers rapid microbial reduction, decreases dependence on chemical disinfectants, and enhances infection control during field visits and public health emergencies [17]. Rechargeable UV sterilization compartments can further strengthen preparedness for communicable disease management and outbreak situations.

Hand Hygiene Integration

Hand hygiene remains the most effective measure for preventing healthcare-associated infections. Modern community nursing bags can support compliance through built-in sanitizer dispensers, portable handwashing kits, touch-free sanitizer systems, soap-and-water modules, and disposable towel compartments [18]. These features are particularly valuable in rural and low-resource settings where access to sanitation facilities may be limited. Some smart nursing bags may also incorporate reminder systems and sensor-based alerts to encourage hand hygiene before and after patient contact. Combined with routine surface disinfection and proper bag placement on clean barriers, these innovations significantly improve infection prevention and patient safety during community-based healthcare delivery. Overall, infection prevention and safety enhancements are essential components of eco-smart community nursing bags, contributing to safer, more efficient, and sustainable field-based nursing practice.

Relevance to community and public health nursing

The modernization of community nursing bags through eco-smart and technology-integrated innovations has significant implications for community and public health nursing practice. Community health nurses serve as frontline healthcare providers delivering preventive, promotive, curative, and rehabilitative services directly within homes and communities. Integration of sustainable materials, portable diagnostic devices, digital documentation systems, telehealth connectivity, and infection-control technologies can improve healthcare accessibility, preparedness, efficiency, and continuity of care in diverse field settings. These innovations are particularly valuable in rural healthcare, disaster response, maternal and child health services, and geriatric home care.

Rural Healthcare

Community nurses working in rural and remote areas often face challenges such as inadequate healthcare infrastructure, transportation barriers, and limited access to specialist services [19]. Technology-integrated nursing bags equipped with portable diagnostic tools, telehealth systems, GPS-enabled navigation, and digital documentation can support point-of-care assessments, chronic disease monitoring, and specialist consultations without requiring patients to travel long distances [20]. Lightweight eco-smart bag designs also improve portability and reduce physical strain for nurses working in geographically challenging environments.

Disaster Response

Community and public health nurses play a vital role in disaster preparedness, emergency response, recovery, and rehabilitation. During disasters such as floods, pandemics, earthquakes, and cyclones, nurses are responsible for triage, emergency care, infection prevention, surveillance, and coordination of healthcare services. Eco-smart nursing bags equipped with solar charging systems, emergency diagnostic tools, telecommunication devices, and infection-control features can enhance preparedness and operational efficiency. These technologies support rapid assessment, mobile documentation, teleconsultation, GPS-based mapping, and real-time disease surveillance, thereby strengthening disaster management and public health emergency response [20,21].

Maternal and Child Health Visits

Maternal and child health services are a major component of community nursing. Community nurses provide antenatal and postnatal care, immunization services, growth monitoring, nutritional counseling, and family health education during home visits and outreach programs [22]. Technology-enabled nursing bags incorporating portable fetal monitoring devices, digital BP monitors, smart thermometers, mobile immunization records, QR-coded maternal records, and teleconsultation support can improve assessment accuracy, documentation, follow-up care, and referral services [23]. Digital reminder systems can further enhance immunization adherence and antenatal follow-up, contributing to improved maternal and neonatal outcomes.

Geriatric Home Care

The growing elderly population has increased the demand for home-based geriatric care services. Community nurses play an important role in chronic disease monitoring, medication management, rehabilitation support, wound care, and health education for older adults [24]. Technology-enabled nursing bags, equipped with portable diagnostic devices, smart medication reminders, remote-monitoring sensors, wearable health devices, fall-detection systems, and telehealth platforms, support continuous monitoring and early identification of health deterioration. AI- and IoT-based systems can track physiological parameters and improve management of chronic illnesses while reducing unnecessary hospital admissions. Lightweight and well-organized eco-smart bags also enhance nurse mobility and support safer, more efficient home-based care. Overall, eco-smart and technology-integrated community nursing bags strengthen healthcare accessibility, quality of care, patient safety, and continuity of services, making them highly relevant to modern community and public health nursing practice [25].

Challenges in implementation

Despite the potential benefits of eco-smart and technology-integrated community nursing bags, several barriers may affect their successful implementation in community healthcare settings. Adoption of advanced nursing technologies depends on adequate financial resources, workforce preparedness, digital competency, healthcare infrastructure, and institutional support. Major challenges include high costs, insufficient training, limited digital literacy, and infrastructural constraints, particularly in low- and middle-income countries [12].

Cost

One of the primary barriers is the high initial investment required for smart healthcare technologies and sustainable materials. Eco-smart nursing bags may incorporate portable diagnostic devices, telehealth equipment, GPS-enabled systems, UV sterilization units, AI-supported monitoring tools, rechargeable batteries, and antimicrobial materials, all of which can significantly increase procurement and maintenance costs [3]. In resource-constrained settings, healthcare organizations often prioritize essential services over digital innovations [8]. Additional expenses related to software updates, equipment maintenance, internet connectivity, data storage, and technical support further increase the financial burden. Therefore, government support and cost-effectiveness evaluations are essential for sustainable implementation.

Training

Successful integration of smart nursing technologies requires adequate education and hands-on training. Community nurses must be competent in operating digital devices, electronic documentation systems, telehealth platforms, infection-control technologies, and data security procedures. However, many nurses may have limited exposure to these technologies during their professional education. Lack of structured training programs, technical support, simulation-based learning opportunities, and continuing education may hinder technology adoption and reduce confidence among healthcare professionals. Continuous capacity-building programs and digital competency training are therefore necessary.

Digital Literacy

Limited digital literacy represents another significant challenge. Community nurses, particularly those working in rural and underserved areas, may experience difficulties using electronic health records, telehealth systems, mobile applications, AI-supported tools, and digital diagnostic devices [12]. Challenges may include operating smart devices, managing electronic records, interpreting digital data, troubleshooting technical issues, and ensuring patient confidentiality. Additionally, patients and community members may have limited digital literacy, which can affect their participation in teleconsultations, mobile health applications, and remote monitoring programs [3]. Strengthening digital health education within nursing curricula and continuing professional development programs is therefore essential.

Infrastructure Limitations

Infrastructure-related barriers remain a major obstacle, especially in rural and low-resource settings. Technology-enabled nursing bags often depend on reliable electricity, internet connectivity, mobile network coverage, technical maintenance services, and digital healthcare systems [12]. Many communities continue to face unreliable power supply, poor internet access, limited technical support, and inadequate healthcare infrastructure, which may affect telehealth services, electronic documentation, AI-assisted monitoring, and synchronization of patient records. Environmental factors such as heat, humidity, dust, and transportation challenges may further affect the durability and performance of electronic devices. Data privacy and cybersecurity concerns also require attention to ensure protection of patient information. Overall, successful implementation of eco-smart community nursing bags requires coordinated efforts in financial planning, workforce training, digital education, infrastructure development, and policy support. Addressing these challenges is essential to ensure equitable, sustainable, and effective integration of smart healthcare innovations into community and public health nursing practice.

Future directions and recommendations

The transformation of traditional community nursing bags into eco-smart and technology-integrated systems represents an important step toward modernizing field-based healthcare delivery. Future developments should focus on strengthening healthcare policies, updating nursing education, promoting research, and developing practical prototypes that address evolving public health needs. Sustainable innovation, digital integration, and interdisciplinary collaboration will be crucial in shaping the future of community health nursing practice [26].

Policy Implications

Successful implementation of eco-smart nursing bags requires supportive healthcare policies and institutional commitment. Policymakers should promote the integration of digital health technologies, telehealth services, portable diagnostic devices, sustainable healthcare procurement, and standardized smart nursing bag designs into community nursing practice [20]. Policies supporting renewable energy utilization, biodegradable healthcare materials, infection-prevention standards, and rural digital healthcare initiatives can further strengthen healthcare delivery. The World Health Organization emphasizes the importance of incorporating digital health competencies and sustainable healthcare approaches to achieve universal health coverage and sustainable development goals [27,28]. Active involvement of nursing leaders in policy formulation is essential to ensure that technological innovations remain practical, safe, and patient-centered.

Nursing Curriculum Updates

Rapid technological advancement has created a need to revise nursing curricula to prepare future nurses for digitally enabled and environmentally sustainable healthcare systems. Nursing education should incorporate digital health competencies, telehealth training, electronic health records, AI and IoT applications, portable diagnostic technologies, infection-prevention innovations, biomedical informatics, and cybersecurity principles. Simulation-based learning and structured training on modernized bag techniques can improve students’ confidence, practical skills, and readiness for technology-driven community practice [29]. Integrating innovation and entrepreneurship concepts into nursing education may also encourage participation in healthcare technology development.

Research Opportunities

Eco-smart and technology-integrated community nursing bags remain an emerging area with substantial research potential. Future studies should focus on design validation, ergonomic assessment, infection-control effectiveness, cost-effectiveness, environmental impact, telehealth outcomes, AI-supported care models, patient safety, and healthcare accessibility in rural settings [2]. Pilot studies and real-world evaluations are needed to assess the effectiveness and acceptability of smart nursing bag technologies. Research should also explore ethical considerations, cybersecurity concerns, digital inclusivity, and sustainability outcomes associated with technology-enabled community healthcare systems.

Prototype Development

Prototype development is essential for transforming innovative concepts into practical healthcare solutions. Collaboration among nurses, engineers, infection-control specialists, public health experts, and technology developers can facilitate the design of user-friendly and efficient smart nursing bags [25]. Future prototypes may incorporate lightweight sustainable materials, antimicrobial surfaces, modular compartments, solar charging systems, UV sterilization units, digital documentation tablets, AI-assisted monitoring tools, IoT-enabled diagnostic devices, QR-code patient management systems, and GPS tracking technologies [25]. Prototype testing should evaluate portability, ergonomic comfort, infection-control effectiveness, durability, battery performance, ease of maintenance, and user satisfaction across diverse healthcare settings. Overall, advancing eco-smart community nursing bags requires coordinated efforts in policy support, educational reform, research, and technological innovation to create sustainable, efficient, and patient-centered solutions for future community healthcare delivery.

## Conclusions

The traditional community nursing bag has long served as both an essential symbol and a functional tool in community health nursing practice. However, rapidly evolving healthcare demands, growing environmental concerns, technological advancements, and shifting public health priorities have highlighted the need to modernize conventional nursing bags into eco-smart, technology-integrated systems. This review emphasizes that incorporation of sustainable materials, portable diagnostic technologies, AI and IoT applications, telehealth systems, infection-prevention innovations, and digital documentation tools can significantly improve efficiency, safety, organization, and quality of field-based nursing care. Eco-smart community nursing bags have the potential to strengthen rural healthcare delivery, disaster response preparedness, maternal and child health services, geriatric home care, and chronic disease management. Despite the promising advantages, challenges related to cost, infrastructure, digital literacy, workforce training, and policy implementation remain important barriers that must be addressed through coordinated efforts from healthcare organizations, policymakers, educators, and technology developers. Future progress requires supportive healthcare policies, updated nursing curricula, interdisciplinary research, and development of practical, user-friendly prototypes tailored to the realities of community healthcare practice. Investment in sustainable and digitally empowered nursing innovations can enhance healthcare accessibility, improve patient outcomes, strengthen infection prevention, and support environmentally responsible healthcare systems. Reimagining the community bag, therefore, represents not merely a technological upgrade but a transformative step toward smarter, safer, sustainable, and patient-centered community health nursing practice for the future.
